# Case report: Bilateral optic nerve sheath meningocele: clinical aspects

**DOI:** 10.3389/fopht.2024.1385485

**Published:** 2024-05-08

**Authors:** Saray Catalán-Coronado, Alba Parrado-Carrillo, Javier Nogués-Castell, Josep Rosinés-Fonoll, Anna Camós-Carreras, Rafel Alcubierre, Maria Teresa Carrión-Donderis, Carolina Bernal-Morales, Bernardo Sánchez-Dalmau

**Affiliations:** ^1^ Institut Clínic d’Oftalmologia, Hospital Clinic, Barcelona, Spain; ^2^ Facultat de Medicina, Universitat de Barcelona, Barcelona, Spain; ^3^ Departament d'Oftalmologia, Hospital del Mar, Barcelona, Spain; ^4^ Departament d'Oftalmologia, Hospital Universitari Mutua de Terrassa, Terrassa, Spain

**Keywords:** optic nerve sheath meningocele, neuroopthalmology, visual field, diplopia, optic nerve

## Abstract

Optic nerve sheath meningocele is an enlargement of the sheath itself, consisting of a collection of cerebrospinal fluid along the perineural space. It should be considered primary if it is not associated with orbital–cerebral neoplasm or with cranio-orbital junction malformations. We report three cases of bilateral primary idiopathic optic nerve sheath meningocele, two of them with gradual vision loss. The first case presented a history of monocular blurred vision of the right eye and headache. It was initially treated with acetazolamide without any improvement, after which optic nerve sheath fenestration was required. The second case showed intermittent binocular diplopia with central 24-2 perimetry defects in the left eye. The third case was first presented as a subacute bilateral conjunctivitis with a suspected orbital pseudotumor. An incidental bilateral optic nerve sheath meningocele was found in the orbital imaging, being totally asymptomatic. In all the cases, orbital and cranial magnetic resonance with contrast and fat suppression was crucial in the diagnosis.

## Introduction

Optic nerve sheath meningocele (ONSM) is an uncommon condition, presented as a primary dilatation of nerve sheath, creating a cavity with cerebrospinal fluid (CSF) around the optic nerve without any underlying pathology (1). Also known as optic nerve dural ectasia, optic hydrops, or CSF cyst, its manifestation varies, from subtle symptoms to progressive vision loss (2). Diagnosis is made with magnetic resonance imaging (MRI) not only to identify the dilatation but also to rule out secondary causes of sheath dilatation, such as inflammation or optic nerve tumors like meningioma, glioma, or arachnoid cysts (3).

Treatment options range according to the broad spectrum of visual repercussion. In some indolent cases, with slow or no progression, observation can be the best option. However, aggressive presentation with progressive visual loss can require surgical treatment such as an optic nerve sheath fenestration (ONSF). Medical treatment with acetazolamide can be considered if intracranial hypertension is associated (4).

We report three different presentations and outcomes of ONSM, one of which has bilateral asymmetric clinical presentation and visual repercussion and two cases were managed conservatively.

## Case 1

**Figure 1:**
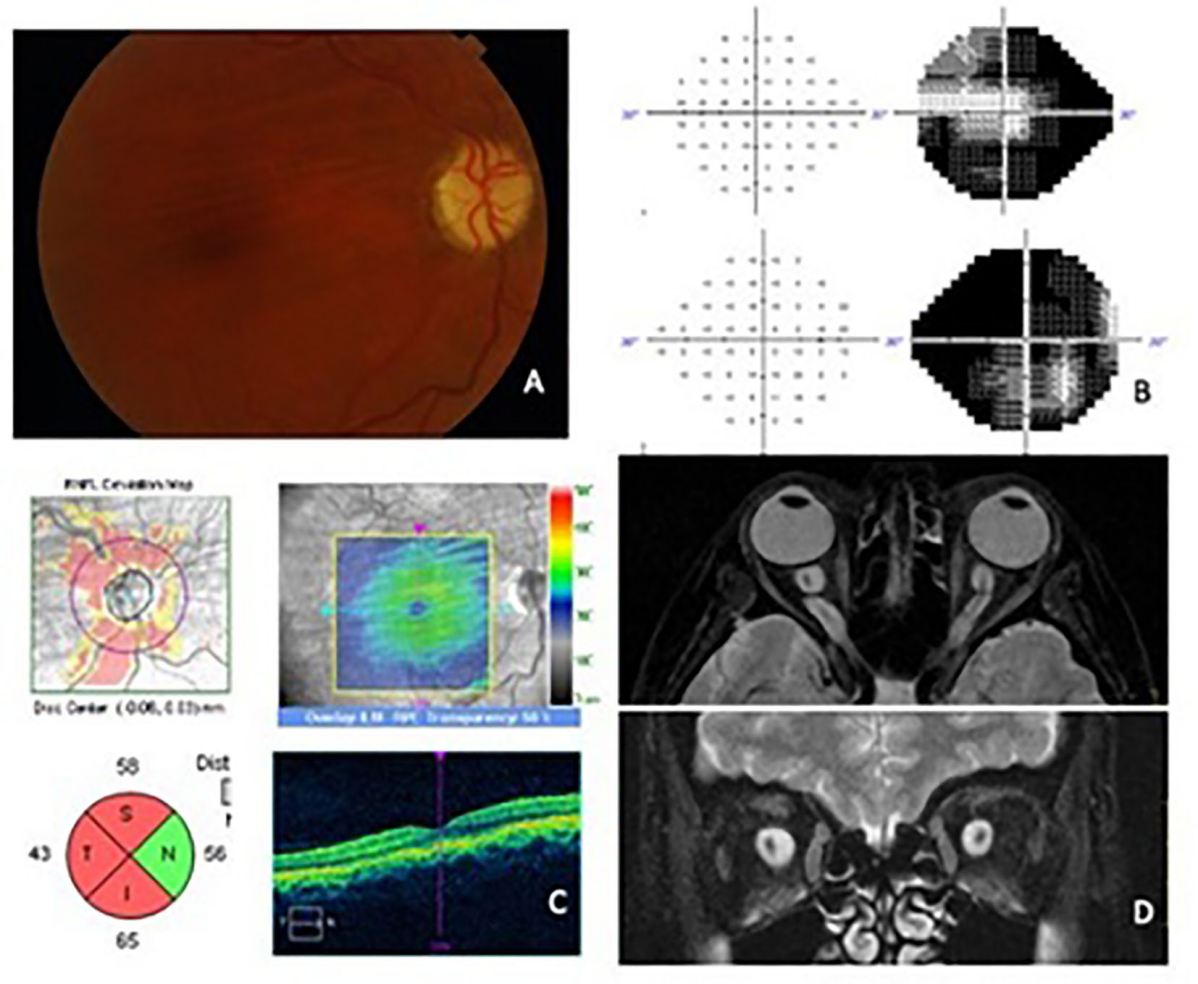
Case 1 (images after bilateral ONSF). **(A)** Right eye fundoscopy showing choroidal folds and optic nerve pallor. **(B)** 24-2 Visual field of the left eye and the right eye. A concentric contraction of the visual fields, more pronounced in the right eye, is shown. **(C)** ONH and macular OCT scans of the right eye with RNFL layer atrophy and choroidal folds in the posterior pole. **(D)** Orbital MRI. Axial and coronal view in T2 with fat suppression showing the marked dilatation of optic nerve sheath in both eyes.

A 43-year-old Caucasian woman presented with a 6-month history of blurred vision in her right eye and headaches. Her past medical history included hypertension, hyperthyroidism, and obstructive sleep apnea syndrome. The best-corrected visual acuity (BCVA) was 6/60 in the right eye and 6/8 in the left eye. A relative afferent pupillary defect (RAPD) was noted in her right eye. Color vision was affected in both eyes with a score of 4/21 in the right eye and 7/21 in the left eye with the Ishihara test plates. Ocular motility was normal and there was no proptosis. The anterior segment examination was normal. Fundoscopy revealed choroidal folds in the right eye and mild optic disc pallor in both eyes. Central 24-2 perimetry (Humphrey–Zeiss field analyzer) showed concentric visual field (VF) reduction in both eyes (**Figure 1**). Orbital and cranial MRI with contrast and fat suppression revealed primary bilateral optic nerve sheath dilatation, leading to the diagnosis of bilateral meningocele. A lumbar puncture was performed to exclude intracranial hypertension. The CSF opening pressure was 180 mmHg with normal cell count and biochemistry.

Medical treatment with acetazolamide 250 mg daily was initiated without any improvement. Because of progressive deterioration of visual function in the right eye, an ONSF was performed, with a gain of visual acuity to 6/30. After a few months, the procedure was also performed in the left eye, without any changes in BCVA but an improvement in color vision with a score on the Ishihara test of 20/21 plates. The patient has been controlled with central 10-2 VF every 6 months and an MRI once per year, without remarkable changes. BCVA remained stable during the 10 years of follow-up.

## Case 2

**Figure 2:**
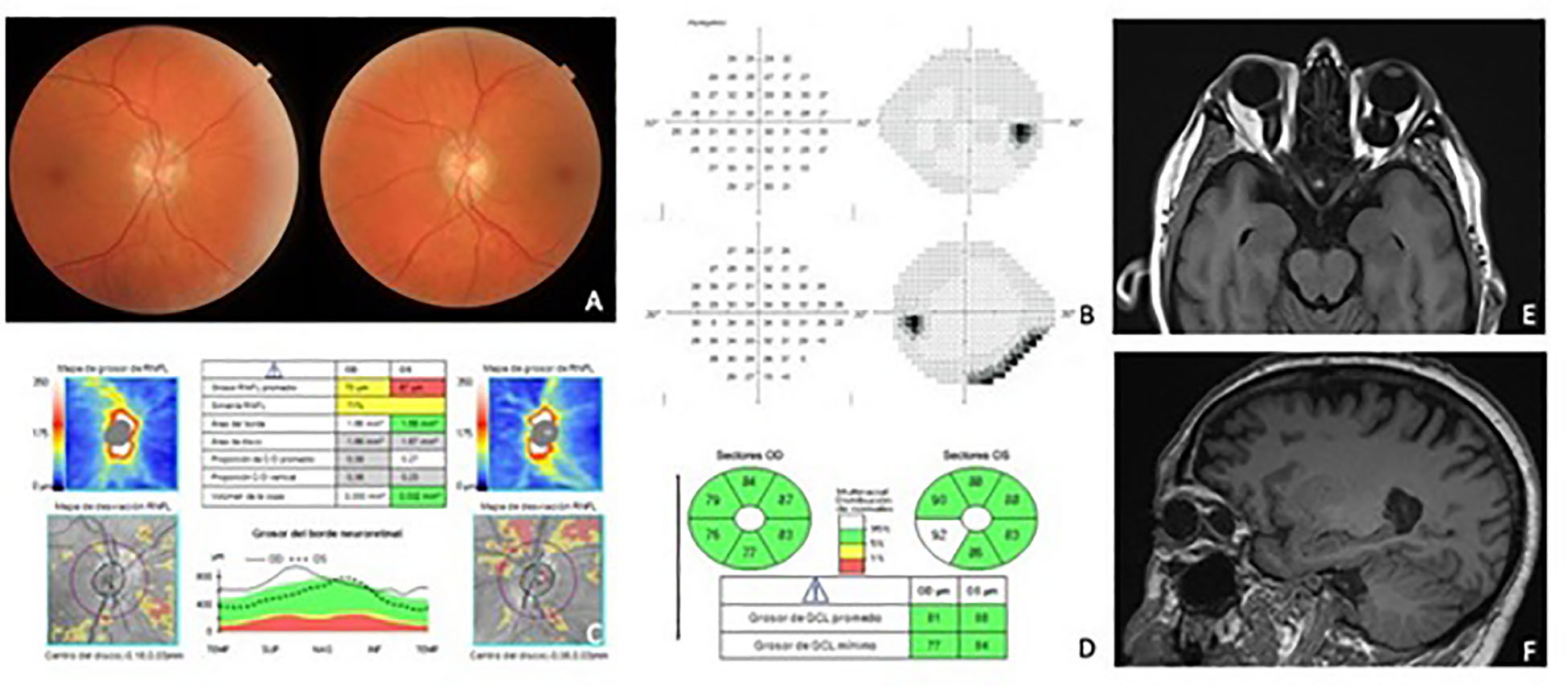
CASE 2 (images from 2023—3 years of follow-up showing no evidence of progression since the diagnosis). **(A)** Right and left eye fundoscopy. **(B)** 24-2 Visual field of both eyes with peripheral inferior nasal defect in the left eye. **(C)** ONH OCT scan. **(D)** GCL sectors of both eyes. **(E)** MRI axial view in T1 dark fluid with bilateral optic nerve sheath dilatation and optic nerve tortuosity. **(F)** Orbital MRI, sagittal view in T1 at the right orbit plane.

A 62-year-old man with no previous medical history presented with intermittent binocular horizontal diplopia. BCVA was 6/7.5 and 6/6 in the right and left eye, respectively. The pupils were isochoric and normorreactive. The Ishihara test score was 21/21 in both eyes. The cover test revealed 35 prism diopters (PD) exotropia and 8 PD hypotropia for the left eye. Funduscopic examination revealed small optic discs with 0.2 cup-to-disc ratio in both eyes with no other findings. Central 24-2 perimetry showed no defects in the right eye but an inferior-nasal defect in the left eye. Ganglion cell optical coherence tomography (OCT) was within normal parameters in both eyes. However, the optic nerve fiber layer OCT revealed a superior defect in the left eye, which was consistent with the VF defect reported. Orbital and cranial MRI were performed, and primary bilateral optic nerve sheath dilatation was identified. The patient has been followed for the past 3 years with no evidence of visual loss or progressive campimetrical loss. The structural OCT scans have also remained stable since the initial diagnosis (**Figure 2**).

## Case 3

**Figure 3:**
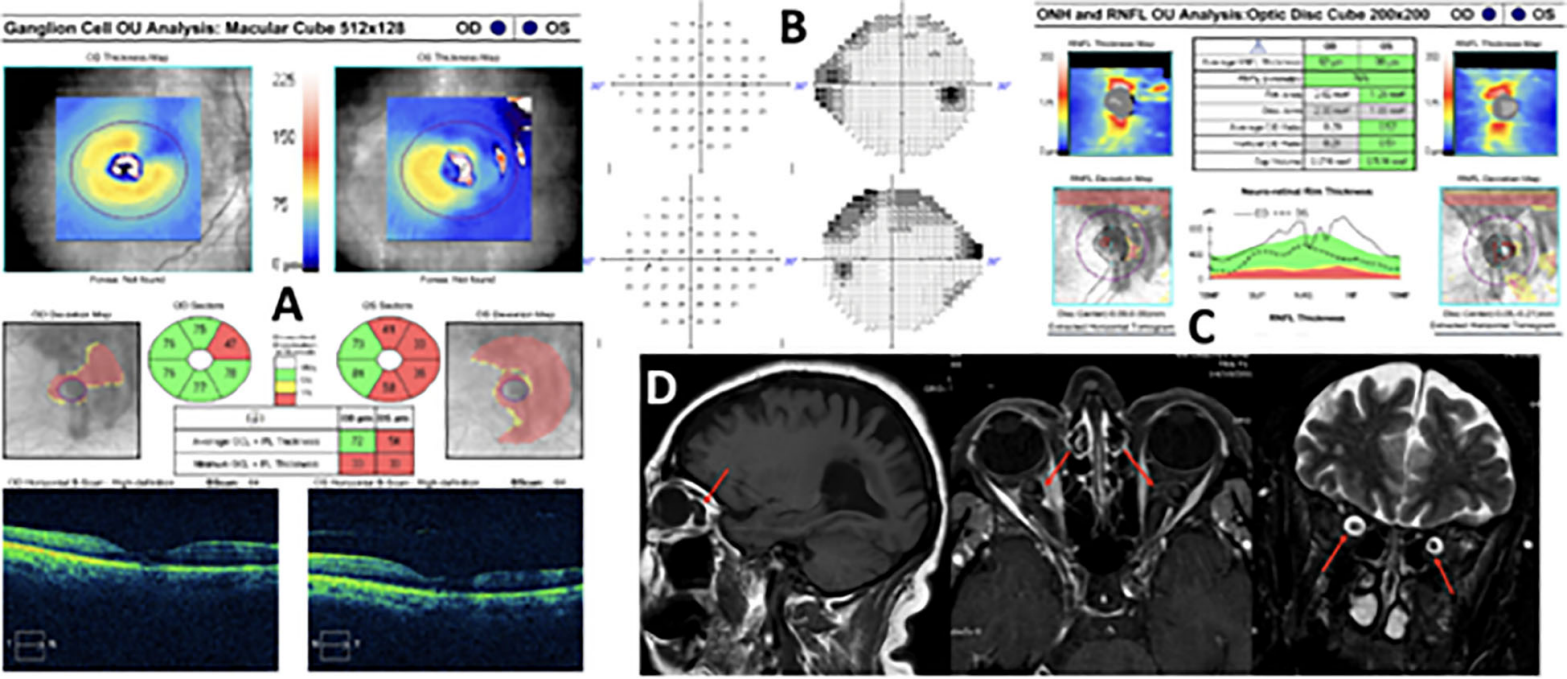
Case 3 (images from 2023—10 years of follow-up showing no progression in the VF, OCTs, or MRI since the initial diagnosis). **(A)** Ganglion cell layer sectors of both eyes. Left eye is artifacted. **(B)** 24-2 Visual field of both eyes with unspecific nasal and superior defects. **(C)** ONH OCT scan shows no optic disc edema, with normal RNFL. **(D)** MRI sagittal (left, T1-weighted), axial (middle, T1-weighted), and frontal (right, T2-weighted) views show bilateral optic nerve sheath dilatation and optic nerve tortuosity (red arrows).

A 70-year-old woman with no ophthalmologic history presented with bilateral subacute conjunctival injection with superficial vascular tortuosity of several weeks’ duration with no improvement using topical corticosteroid treatment. Ocular examination revealed conjunctival hyperemia, mild episcleral vascular tortuosity, and chemosis with a subtarsal papillary reaction. Baseline BCVA was 6/7.5 in both eyes. Intraocular pressure was 20 mmHg in both eyes. Neuro-ophthalmic examination revealed no pathological findings. An orbital CT scan was performed to exclude an orbital pseudotumor, which incidentally showed thickening of both optic nerves. A VF and a brain MRI with gadolinium and fat suppression were performed to complete the evaluation. The MRI showed increased sheath size and tortuosity and kinking of both intraorbital optic nerves associated with increased CSF (**Figure 3**). An empty sella turcica was also described in the report. The diagnosis was idiopathic bilateral ONSM without clinical or other signs of intracranial hypertension.

The patient BCVA remained stable in both eyes throughout the follow-up period. The VF showed no significant findings with a high number of false positives. Neither macular nor papillary OCT showed any changes. The patient underwent cataract surgery in both eyes with a final BCVA of 6/6 10 years after the initial ONSM diagnosis.

## Discussion

The “optic nerve sheath meningocele*”* (5) was first introduced in Garrity and colleagues’ landmark paper in which 13 patients were described (1). Since then, a few cases have been described (4).

Presenting symptoms of ONSM include visual disorders like blurred vision or VF disturbances, refractive error (hyperopic shift), headache, and retroorbital pain. Signs at the time of diagnosis can be varied and include proptosis, choroidal folds, disc swelling, or pale disc and cystoid macular edema (5). One of our cases presented with subacute conjunctival chemosis, hyperemia, and episcleral vascular tortuosity, not described before for ONSM. We believe that this was due to the orbital intrinsic compression effect of ONSM that may raise the venous outflow resistance producing chemosis and episcleral vessels’ tortuosity. This process has already been described in other orbital pathologies such as compressive orbital lesions, idiopathic orbital inflammation, or thyroid eye disease (6).

The diagnosis is confirmed by MRI that reveals the optic nerve sheath dilatation but normal-sized optic nerves (“bull’s eye” appearance on coronal T2-weighted images) (4). Fat-suppression techniques are appropriate to rule out intraorbital tissue lesions or optic nerve compression. It is also recommended to perform a lumbar puncture to determine the CSF opening pressure to exclude idiopathic intracranial hypertension. Related VF defects include enlargement of the blind spot, arcuate field defects, and concentric constriction (4, 7). It is important to periodically assess the VF defects’ progression, as sometimes small peripheral defects can be stable for years without further intervention (as shown in case 2). On the other hand, other patients may have progressive loss of the VF that requires more aggressive management.

Among published reports, six cases of ONSM have been bilateral. Shanmuganathan et al. in 2002 published a case with unilateral macular edema (8). In 2017, Mahatma presented a bilateral ONSM case with unilateral vision loss in the right eye. Berker et al. described a patient with a pale optic nerve in the right eye and chronic disc edema in the left eye. In all cases, the findings were asymmetrical although the affectation was bilateral. In the past 2 years, three more cases of bilateral ONSM have been published (4, 9, 10).

The etiology of ONSM is still uncertain. It has been suggested that congenital narrowing of the optic canal or congenital cranio-orbital junction abnormalities could cause a sheath dilatation by altering the CSF flow within the periorbital subarachnoid space (3). Another theory postulates that a difference in osmotic gradient between the cerebral and perioptic subarachnoid space may be involved. The role of intracranial pressure is unclear, as only a few of the reported patients have undergone a lumbar puncture (7).

Because ONSM is an uncommon pathology, there is a lack of agreement in favor of one treatment option over another. Medical therapy with oral acetazolamide has shown good results in mild cases (4, 8). However, it is widely accepted that ONSF should be performed when there is evidence of decreased BCVA or VF progression loss. Case 1 was the only one of our patients to undergo a bilateral ONSF.

## Conclusions

We present three cases of ONSM followed up in our center. All three had different manifestations and visual repercussions, highlighting the variable presentation of this pathology.

The pathophysiology of ONSM remains unclear. MRI of the orbits helps to diagnose the dilatation in patients with bilateral optic neuropathies and VF loss; however, there is no evidence of a direct correlation between the degree of dilatation and the optic nerve repercussion. Thus, there is still no way of predicting the course of the disease or the long-term visual consequences. In some cases of ONSM, the progression can be severe and more aggressive management of the pathology may be required such as proceeding with an ONSF.

We encourage ophthalmologists to perform a close monitoring of these patients, especially in the first years after diagnosis.

## Data availability statement

The original contributions presented in the study are included in the article/supplementary material. Further inquiries can be directed to the corresponding author.

## Ethics statement

Written informed consent was obtained from the individual(s) for the publication of any potentially identifiable images or data included in this article.

## Author contributions

SC-C: Writing – original draft, Writing – review & editing, Methodology. AP-C: Writing – original draft. JN-C: Methodology, Writing – original draft, Writing – review & editing. JR-F: Methodology, Writing – original draft, Writing – review & editing. AC-C: Writing – review & editing. RA: Writing – review & editing. MC: Writing – original draft. CB-M: Writing – review & editing. BS-D: Conceptualization, Writing – review & editing.
